# Machine Learning-Based Prediction of Comprehensive Lipid Response to Dietary Interventions in Overweight and Obese Women

**DOI:** 10.3390/nu18121974

**Published:** 2026-06-18

**Authors:** Shula Shazman

**Affiliations:** Department of Mathematics and Computer Science, The Open University of Israel, Ra’anana 4353701, Israel; shulash@openu.ac.il

**Keywords:** personalized nutrition, machine learning, dyslipidemia, cardiovascular risk, dietary interventions, obesity, triglycerides, lipoproteins, predictive modeling, women’s health

## Abstract

**Background**: Inter-individual variability in lipid response to dietary interventions complicates cardiometabolic prevention in overweight and obese women. Although several dietary strategies improve lipid profiles on average, predictors of comprehensive, multi-marker lipid improvement remain unclear. **Objective**: To identify baseline clinical predictors of comprehensive lipid improvement across seven dietary interventions and to evaluate the performance of three machine learning (ML) classifiers in predicting a composite Global Score. **Methods**: This secondary analysis pooled individual-level data from 284 overweight or obese women enrolled in three randomized controlled trials (RCTs). Participants were assigned to continuous energy restriction (CER), intermittent energy restriction (IER), intermittent energy and carbohydrate restriction (IECR), IECR with added protein and fat (IECR+PF), high-carbohydrate, high-monounsaturated-fat, or daily energy-restriction diets. Eleven baseline clinical features served as predictors. Four binary lipid improvement scores (TC/HDL, LDL/HDL, non-HDL cholesterol, TG/HDL) were calculated from baseline to week 12, and a composite Global Score was defined as TRUE only when all four improved. Three ML classifiers (J48, Logistic Model Tree [LMT], Random Forest) were evaluated using stratified 10-fold cross-validation. **Results**: Overall, 30–35% achieved improvement in the Global Score. Improvement rates varied across diets, with High Mono and High Carb showing the highest rates (48.4%). LMT performed best (AUC = 0.66; accuracy = 70%). Baseline TG, BMI, age, total cholesterol, and weight were the strongest predictors. **Conclusions**: Comprehensive lipid improvement varies across dietary strategies and is influenced by baseline triglycerides, adiposity, age, and diet type. ML-based stratification may support personalized dietary prescriptions.

## 1. Introduction

Cardiovascular disease prevention increasingly depends on the ability to identify which individuals are most likely to benefit from specific dietary interventions, particularly among women at elevated metabolic risk.

Dyslipidemia—characterized by elevated low-density lipoprotein (LDL) cholesterol, reduced high-density lipoprotein (HDL) cholesterol, elevated triglycerides (TG), and unfavorable lipoprotein ratios—is a primary modifiable risk factor for cardiovascular disease (CVD), the leading cause of morbidity and mortality in high-income countries [1,2]. Converging evidence from genetic, epidemiological, and clinical studies has established that atherogenic lipoproteins—particularly LDL cholesterol and TG-rich lipoprotein remnants—are causally linked to atherosclerotic CVD [3,4]. Beyond individual lipid parameters, composite lipid ratios such as TC/HDL and LDL/HDL capture the balance between atherogenic and atheroprotective lipoprotein fractions and have consistently outperformed individual markers in predicting cardiovascular events across diverse populations, including women [5,6]. The TG/HDL ratio further serves as a clinically accessible surrogate for insulin resistance and the atherogenic small, dense LDL phenotype [7,8]. Among women, the transition through midlife is associated with adverse shifts in lipid profiles, including rising LDL cholesterol and TC/HDL ratios, that substantially increase long-term cardiovascular risk [9]; sex-specific analyses underscore the importance of proactive lipid management during the perimenopausal period, when hormonal changes accelerate atherogenic lipid remodeling [10,11]. Dietary intervention remains a cornerstone of lipid management, yet the magnitude and pattern of lipid response vary considerably across individuals and intervention types [12,13].

A growing body of evidence suggests that energy restriction strategies differ not only in their effects on weight but also in their downstream impact on lipid metabolism. Intermittent energy restriction (IER), which alternates periods of energy restriction with normal intake, has attracted considerable interest as an alternative to continuous energy restriction (CER) [14,15]. Whereas CER requires sustained daily caloric restriction, IER leverages periodic metabolic adaptations—including enhanced fatty acid oxidation, reduced hepatic lipogenesis, and improved insulin sensitivity—that may confer distinct lipid benefits beyond those attributable to weight loss alone [16,17,18]. Macronutrient composition—particularly dietary fat quality and carbohydrate content—also independently influences lipid outcomes [19,20] with high-monounsaturated-fat and high-carbohydrate diets producing distinct effects on LDL, TG, and HDL [19,21,22]. Despite this evidence, most published analyses assess average group-level responses rather than addressing why some individuals achieve comprehensive lipid improvement while others do not.

The concept of personalized nutrition—tailoring dietary recommendations to individual clinical, metabolic, and behavioral characteristics—has gained momentum as a framework for improving the precision and efficacy of dietary interventions [23,24,25,26,27,28].

Recent advances in precision nutrition have further highlighted the contribution of genetic variation, gut microbiome composition, metabolomic profiles, and environmental exposures to inter-individual differences in dietary response [27,29]. Multi-omics approaches combined with artificial intelligence are increasingly being explored to identify responder phenotypes and optimize dietary recommendations [30]. However, despite their scientific promise, these approaches often require specialized data collection and analytical infrastructure that may limit their routine clinical applicability. Consequently, there remains substantial interest in determining whether readily available clinical variables can provide useful predictive information for personalized dietary interventions.

Machine learning (ML) methods are increasingly applied in clinical medicine and nutrition research to identify complex, non-linear relationships between baseline characteristics and treatment outcomes that conventional statistical approaches may not detect [31,32,33].

Recent studies have applied ML approaches to predict glycemic responses, weight-loss trajectories, and other metabolic outcomes following dietary interventions. Nevertheless, many existing prediction models rely on genomic, microbiome, metabolomic, or other high-dimensional datasets [30]. Comparatively fewer studies have examined whether clinically accessible baseline variables alone can predict lipid responsiveness across multiple dietary intervention strategies. This represents an important knowledge gap because clinical variables are widely available and readily translatable to routine practice.

Decision tree algorithms—including J48, a widely used implementation of the C4.5 induction algorithm [34], Logistic Model Trees (LMT) [35], and Random Forest [36]—offer interpretable or ensemble-based alternatives to black-box models by generating explicit clinical decision rules that can be meaningfully translated into practice [37].

To date, no study has applied ML classification to predict comprehensive, multi-marker lipid improvement across multiple dietary intervention strategies simultaneously. Existing prediction models have largely focused on weight loss outcomes or single lipid parameters and have rarely been developed in the context of pooled, multi-trial data [38,39]. Robust development and reporting of multivariable prediction models require adherence to established methodological guidelines [40]. The identification of baseline clinical predictors that distinguish likely responders from non-responders could meaningfully inform dietary prescription and support more targeted lipid management in overweight and obese women, in whom adiposity-related metabolic dysregulation amplifies inter-individual variability in dietary lipid response [41]. Furthermore, improvements in individual lipid markers do not necessarily occur in parallel and may reflect different aspects of cardiometabolic risk [42]. Therefore, evaluating comprehensive lipid improvement across multiple lipid parameters may provide a broader assessment of metabolic benefit than focusing on any single marker alone. This rationale motivated the development of the composite Global Score used in the present study, which required simultaneous improvement across four clinically relevant lipid outcomes.

The present study sought to evaluate inter-individual variability in lipid responses to dietary interventions and to develop clinically interpretable predictive models for personalized nutrition. Specifically, we aimed to: (1) quantify the prevalence of favorable changes in individual lipid markers and in a composite measure of comprehensive lipid improvement among overweight and obese women participating in seven dietary intervention strategies derived from three randomized controlled trials; (2) assess and compare the performance of three machine learning algorithms—J48 Decision Tree, Logistic Model Tree (LMT), and Random Forest—in predicting comprehensive lipid responsiveness; (3) identify the baseline clinical characteristics most strongly associated with treatment success; and (4) examine the decision pathways generated by the most interpretable model to provide clinically meaningful insights into determinants of dietary lipid response.

## 2. Materials and Methods

### 2.1. Study Population and Dataset

This study presents a secondary analysis of pooled individual participant data from three previously published randomized controlled trials [14,15,22]. The combined dataset comprised 285 records from overweight or obese women; one participant was excluded due to missing data on baseline lipid values, yielding a final analytic sample of 284 participants. Participants were allocated to one of seven dietary interventions: continuous energy restriction (CER, n = 53), intermittent energy restriction (IER, n = 54), intermittent energy and carbohydrate restriction (IECR, n = 37), IECR supplemented with ad libitum protein and fat (IECR+PF, n = 38), a high-carbohydrate weight-loss diet (High Carb, n = 31), a high-monounsaturated-fat diet (High Mono, n = 31), and daily energy restriction with a ~20% deficit (DER, n = 40). Mean age was 44.5 ± 8.0 years (range 19–69) and mean BMI was 31.7 ± 5.2 kg/m^2^ (range 22.3–45.6). Baseline anthropometric and biochemical characteristics by intervention group are presented in Table 1A,B. Significant baseline differences across intervention groups were observed for age, weight, BMI, hip circumference, WHR, LDL cholesterol, TG, and fasting glucose (all *p* ≤ 0.043; Table 1A,B). These differences reflect the multi-trial pooling design rather than randomisation failure within a single study, and introduce potential residual confounding that cannot be fully eliminated through ML-based feature adjustment alone; between-group comparisons of lipid improvement rates should therefore be interpreted with appropriate caution.

### 2.2. Baseline Features

**Figure 1:**
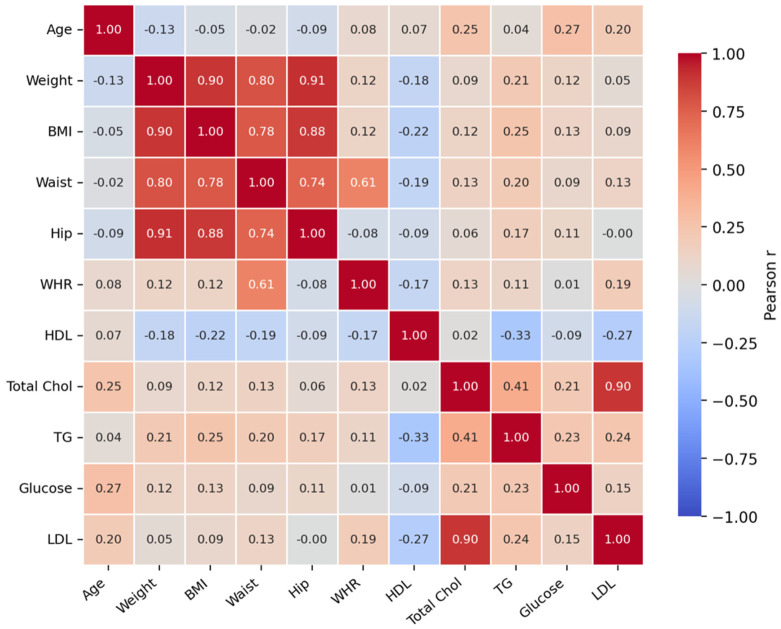
Pearson Correlation Heatmap of Baseline Clinical Features (n = 284). Color intensity reflects the magnitude of the Pearson correlation coefficient, ranging from deep blue (strong negative correlation, r = −1.00) through white (no correlation, r = 0) to red (strong positive correlation, r = +1.00). Strong positive correlations were observed among body composition variables—weight, BMI, waist circumference, and hip circumference (r = 0.74–0.91). Total cholesterol and LDL were strongly positively correlated (r = 0.90), and HDL showed a negative correlation with TG (r = −0.33), consistent with established metabolic relationships. WHR showed a distinct pattern, correlating moderately with waist circumference (r = 0.61) but negligibly with hip circumference (r = −0.08) and overall weight (r = 0.12). All 11 baseline features were retained as model inputs.

For each of the 284 participants, 11 baseline clinical features were extracted from the pooled dataset to serve as model inputs: age, body weight, BMI, waist circumference, hip circumference, WHR, HDL, total cholesterol, TG, fasting glucose, and LDL. Pearson correlation heatmap was computed (Figure 1). Strong positive correlations were observed among weight, BMI, waist circumference, and hip circumference (r = 0.82–0.93). Total cholesterol and LDL were strongly positively correlated (r = 0.89), and HDL showed a notable negative correlation with TG (r = −0.31), consistent with established metabolic relationships. These correlation patterns were considered in model interpretation, and all 11 predictors were retained for model training.

### 2.3. Outcome Variable Definition

To define treatment response, outcome variables were constructed from changes in lipid-related parameters between baseline and week 12. Four binary lipid improvement scores were derived: (1) TC/HDL ratio difference [TC/HDL(baseline) − TC/HDL(week 12)]; (2) LDL/HDL ratio difference [LDL/HDL(baseline) − LDL/HDL(Week 12)]; (3) non-HDL cholesterol difference [non-HDL(baseline) − non-HDL(Week 12)]; and (4) TG/HDL ratio difference [TG/HDL(baseline) − TG/HDL(Week 12)]. Each score was coded TRUE when the direction of change indicated improvement relative to baseline, and FALSE otherwise. A composite Global Score was defined as TRUE only when all four individual scores were simultaneously TRUE, thereby identifying participants who achieved comprehensive improvement across all assessed lipid parameters. The Global Score is an exploratory composite endpoint constructed for this analysis and has not been independently validated; findings based on this outcome should be interpreted accordingly and confirmed in prospective studies using pre-specified composite lipid endpoints.

**Figure 2:**
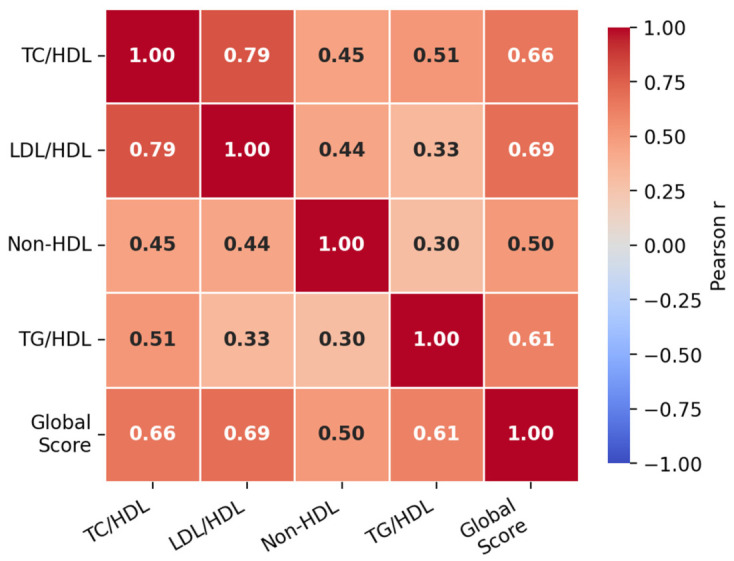
Pearson Correlation Heatmap of Outcome Variables and Composite Global Score (n = 284). Color intensity reflects the magnitude of the Pearson correlation coefficient, ranging from deep blue (negative correlation) through white (no correlation) to red (positive correlation). All pairwise correlations were positive, indicating that improvement in one lipid parameter was generally associated with improvement in others. The strongest correlation was observed between TC/HDL and LDL/HDL improvement scores, reflecting their shared biological and mathematical components. The TG/HDL improvement score showed comparatively weaker associations with the remaining scores, supporting its contribution as a relatively independent dimension of lipid response. The Global Score, defined as TRUE only when all four component scores were simultaneously TRUE, was positively correlated with each individual score, confirming its role as a composite indicator of comprehensive lipid improvement.

Pearson correlations among the four binary scores and the Global Score were examined prior to modelling (Figure 2). Moderate positive correlations were observed among TC/HDL, LDL/HDL, and non-HDL improvement scores, with the strongest association between TC/HDL and LDL/HDL—consistent with the mathematical and biological overlap between these measures. The TG/HDL improvement score showed weaker correlations with the other three scores, supporting its inclusion as a relatively independent dimension of lipid response. The Global Score was positively correlated with all four component scores, confirming that it captures a more stringent criterion for overall lipid improvement rather than redundantly reflecting any single marker.

### 2.4. Machine Learning Classifiers and Evaluation

Three supervised classification algorithms were trained to predict the Global Score: (1) a J48 decision tree (C4.5 implementation), which produces interpretable if–then rules; (2) a Logistic Model Tree (LMT), combining decision tree induction with logistic regression at the leaves; and (3) a Random Forest ensemble of 100 trees using bootstrap aggregation. Model performance was evaluated using stratified 10-fold cross-validation. Reported metrics include AUC, overall accuracy, F1-score, recall, and precision. Random Forest feature importance scores were used to rank baseline predictors by their relative contribution to classification. Between-group differences in weight change were assessed using the Mann–Whitney U test; a two-sided *p* < 0.05 was considered statistically significant. While stratified 10-fold cross-validation was employed to reduce overfitting risk, it is acknowledged that the per-intervention subgroup sizes (range n = 31–54) are modest relative to the complexity of the seven-group classification problem, and that internal cross-validation does not substitute for external validation in an independent cohort. All subgroup findings should therefore be interpreted as hypothesis-generating and require replication in larger independent datasets prior to clinical application.

## 3. Results

### 3.1. Improvement Rates Across Individual Lipid Scores

**Figure 3:**
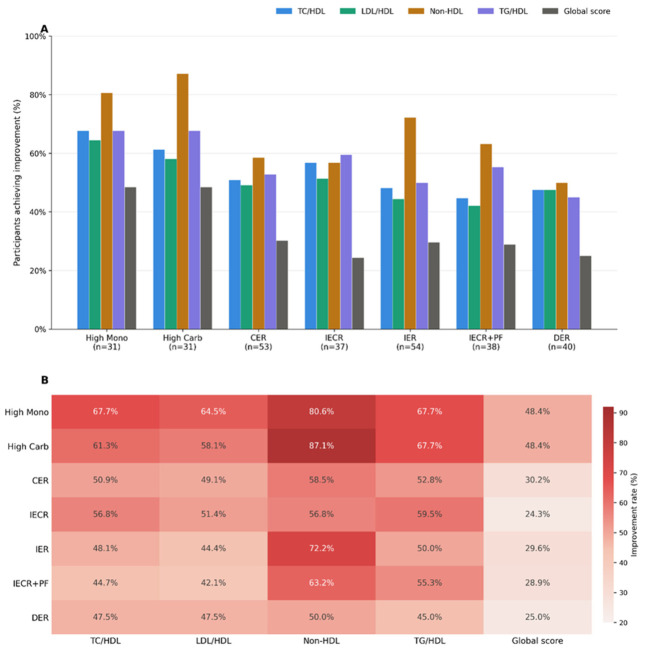
Lipid improvement rates (%) across individual scores and composite Global Score, stratified by dietary intervention. (**A**) Grouped bar chart displaying the percentage of participants achieving improvement in each of four lipid parameters—TC/HDL ratio, LDL/HDL ratio, non-HDL cholesterol, and TG/HDL ratio—and in the composite Global Score, by dietary intervention group. (**B**) Heatmap representation of the same data; color intensity reflects improvement rate, with darker shading indicating higher proportions of improvers. The Global Score was defined as TRUE only when all four individual lipid improvement scores were simultaneously TRUE. Improvement was defined as a favorable change from baseline to week 12 in the respective lipid parameter. CER, continuous energy restriction; IER, intermittent energy restriction; IECR, intermittent energy and carbohydrate restriction; IECR+PF, IECR supplemented with ad libitum protein and fat; High Carb, high-carbohydrate weight-loss diet; High Mono, high-monounsaturated-fat diet; DER, daily energy restriction (~20% deficit); TC, total cholesterol; HDL, high-density lipoprotein; LDL, low-density lipoprotein; TG, triglycerides.

The overall rate of comprehensive lipid improvement (Global Score = TRUE) was 32.3% (92 of 284 participants). Across the full cohort, the four individual lipid improvement scores showed markedly different rates of attainment (Figure 3A). Non-HDL cholesterol was the most frequently improved parameter (65.8% of women), followed by the TG/HDL ratio (55.6%), TC/HDL ratio (52.8%), and LDL/HDL ratio, which was the hardest to improve (50.0%). The composite Global Score, requiring simultaneous improvement across all four parameters, was achieved by only 32.3% of participants, reflecting the stringency of the combined criterion.

Stratification by dietary intervention revealed notable differences in lipid response profiles (Figure 3A,B; Table 2). High Mono and High Carb yielded the highest Global Score rates (both 48.4%), consistently ranking first or second across all four individual scores. High Mono achieved the highest TC/HDL (67.7%) and LDL/HDL (64.5%) improvement rates, while High Carb led on Non-HDL cholesterol improvement (87.1%). The heatmap (Figure 3B) further illustrates that these two macronutrient-focused diets produced uniformly darker shading across all lipid parameters, contrasting with the comparatively lighter profile of DER, which yielded the lowest improvement rates across all four scores. Notably, IER demonstrated an uneven response pattern—despite a relatively high Non-HDL improvement rate (72.2%), it yielded the lowest LDL/HDL improvement rate among all interventions (44.4%), which constrained its overall Global Score performance (29.6%).

All seven dietary interventions produced statistically significant reductions in waist and hip circumference from baseline to Week 12 (all *p* < 0.001, Table 3 and Table 4). The greatest waist reductions were observed in the High Carb (−6.74 ± 4.34 cm) and High Mono (−6.63 ± 4.47 cm) groups, compared with more modest reductions in DER (−3.47 ± 4.18 cm) and IER (−3.74 ± 4.29 cm); between-group differences were statistically significant (F(6,277) = 3.02, *p* = 0.007). Hip circumference reductions were most pronounced in High Mono (−8.63 ± 4.50 cm) and High Carb (−7.97 ± 3.58 cm) groups, with highly significant between-group variation (F(6,277) = 11.65, *p* < 0.001). In contrast, no dietary group produced a significant change in WHR (all *p* > 0.05), and between-group differences were non-significant (F(6,277) = 1.16, *p* = 0.331), suggesting that the macronutrient-focused and energy-restriction protocols reduced absolute circumferences proportionally rather than selectively targeting central versus peripheral adiposity.

### 3.2. Machine Learning Classifier Performance

Three classifiers were evaluated for the prediction of the composite Global Score using 10-fold cross-validation (Table 5). The LMT classifier outperformed both comparators across all five metrics (AUC = 0.66, accuracy = 70%, F1-score = 0.67, recall = 0.70, precision = 0.68). Random Forest achieved intermediate performance (AUC = 0.62, accuracy = 68%), while J48 yielded the lowest AUC (0.58), though its accuracy (65%) and F1-score (0.65) were comparable to Random Forest. All three models exceeded the 50% random-chance baseline.

Given the class imbalance in the outcome variable (Global Score = TRUE in 32.3% of participants), accuracy alone may overestimate predictive performance; F1-score and recall were therefore considered the primary metrics of interest. Under this criterion, LMT demonstrated the most clinically relevant performance, correctly identifying 70% of true improvers (recall = 0.70) while maintaining acceptable precision (0.68). The AUC values across all models were modest (range 0.58–0.66), indicating limited but above-chance discriminative ability, which should be interpreted in the context of the relatively small sample size, the heterogeneity of the pooled dataset, and the inherent difficulty of predicting individualized dietary response from baseline clinical features alone. Formal significance testing between classifier performance metrics was not conducted; observed differences should therefore be interpreted with caution.

### 3.3. Feature Importance

Random Forest feature importance analysis revealed a relatively even distribution of predictive weight across all 11 baseline variables, indicating that no single clinical marker dominated the model’s decision process. The five highest-ranked features were baseline TG (9.7%), BMI (9.6%), age (9.0%), total cholesterol (9.0%), and body weight (8.8%), whereas HDL carried the lowest individual importance (6.0%). This pattern suggests that comprehensive prediction of lipid improvement requires integrating multiple anthropometric and biochemical indicators rather than relying on any isolated variable.

**Figure 4:**
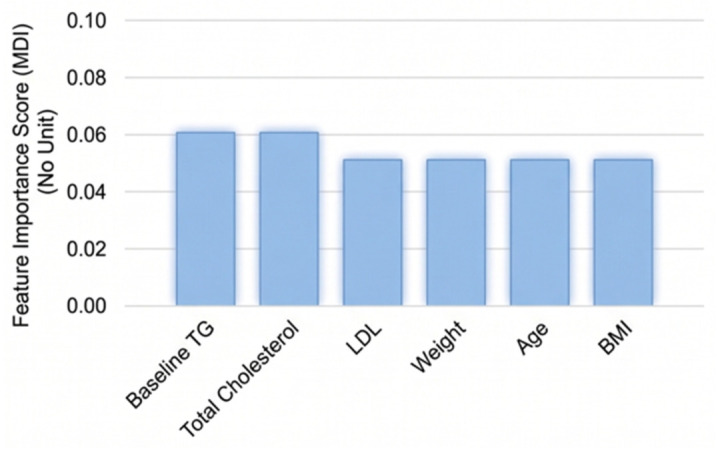
Histogram of Random Forest Feature Importance Values Mean Decrease in Impurity (MDI). Histogram displaying the distribution of Random Forest feature importance scores (MDI) across the 11 baseline predictors. Each bar represents the raw decimal score of mean decrease in impurity (MDI) for a single feature, contributing to the model’s overall predictive performance. We observe that the predictors TG and total cholesterol show the highest importance value (0.06).

To visualize these relative contributions, a histogram of feature importance values was generated (Figure 4). The distribution highlights the modest but meaningful variability across predictors, with triglycerides (TG) emerging as the most influential feature, followed closely by total cholesterol, age, BMI and body weight. Features such as HDL, WHR, and fasting glucose appear at the lower end of the distribution but still contribute non-negligible information to the ensemble model. The relatively uniform height of the bars illustrates that the model relies on a multi-feature clinical signature rather than a dominant biomarker. The overall shape of the histogram reinforces the interpretation that the Random Forest classifier leverages a broad clinical profile, consistent with the multifactorial nature of lipid metabolism and dietary response.

### 3.4. Age-Group Analysis

Women aged 45 or older achieved comprehensive cholesterol improvement at nearly twice the rate of younger women (42.9% vs. 24.8%). This pattern persisted across all three BMI categories (Table 6), with the 45+ group consistently outperforming the ≤44 group within each BMI stratum. The most pronounced absolute difference was in BMI 30–35 (51.4% vs. 32.1%), while the smallest was in BMI >35 (30.0% vs. 17.8%), suggesting that severe obesity may partially attenuate the age-related advantage.

Dietary intervention moderates the age effect. Among women aged 45+, IER yielded a Global Score rate of 60.0%, followed by High Carb (66.7%) and CER (50.0%). High Mono was the only intervention where the ≤44 group marginally outperformed the 45+ group (53.8% vs. 44.4%), a pattern that warrants replication in a larger sample.

A formal multivariate logistic regression incorporating an Age × BMI interaction term confirmed these patterns and is reported in Table 7.

### 3.5. BMI-Group Analysis

To explore how baseline adiposity influences metabolic responsiveness, participants were stratified into three BMI categories and Global Score outcomes were compared across groups. A non-linear relationship between BMI and success rate was observed: women with intermediate obesity (BMI 30–35 kg/m^2^) achieved the highest Global Score rate (40.0%), followed by the overweight group (BMI 25–30 kg/m^2^, 32.8%), whereas severe obesity (BMI >35 kg/m^2^) was associated with the lowest rate (22.7%).

This inverted-U pattern suggests that moderate obesity may confer greater metabolic flexibility—potentially reflecting more efficient lipid turnover, adaptive mitochondrial function, and balanced inflammatory signalling—allowing for more pronounced improvement within the 12-week intervention period. In contrast, women with BMI >35 kg/m^2^ may experience metabolic rigidity and impaired lipid mobilisation, limiting their capacity for comprehensive lipid improvement. These findings underscore the need for BMI-stratified approaches when designing metabolic interventions.

### 3.6. Baseline Cholesterol Level as a Predictor of Response

Women with high baseline total cholesterol (>6.0 mmol/L, n = 65) showed a markedly higher Global Score rate compared to women with normal-range values (46.2% vs. 28.3%). This effect was most pronounced for CER and IER (both +38 percentage points), consistent with a floor effect whereby greater initial lipid burden provides more margin for proportional improvement. The effect was absent for IECR+PF, where women with high baseline cholesterol performed similarly to those with normal values (25.0% vs. 30.8%, Table 7).

### 3.7. Relationship Between Weight Loss and Cholesterol Improvement

**Figure 5:**
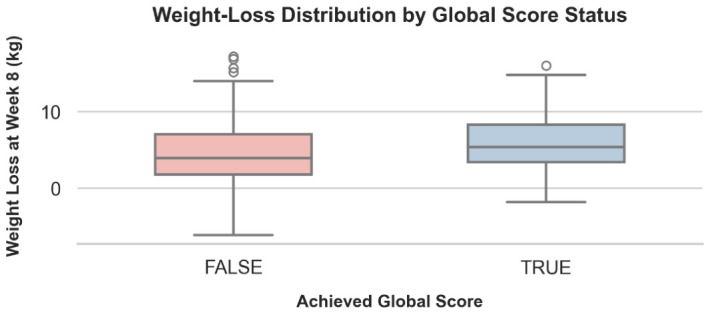
Weight-loss distributions at week 8 in TRUE vs. FALSE Global Score groups. Boxplots showing the distribution of weight loss at week 8 among women who achieved the 12-week Global Score (TRUE) compared with those who did not (FALSE). Weight-loss outcomes are presented for week 8 because body weight was not measured at the 12-week follow-up. Although the TRUE group shows greater average weight reduction by the end of the weight-tracking period, the wide overlap between distributions indicates that long-term lipid improvement is not solely dependent on weight loss magnitude. Outliers represent individuals with unusually high or low weight-loss responses.

To evaluate the relationship between weight reduction and metabolic improvement, weight-loss outcomes were compared between women who achieved the Global Score and those who did not. While metabolic indicators were tracked across the full 12-week study duration, weight measurements were specifically evaluated at week 8, as weight data were not collected at the 12-week time point. As illustrated in Figure 5, women in the TRUE group experienced greater overall weight loss by week 8 compared with the FALSE group. Despite this difference in mean weight reduction, the distributions showed substantial overlap, indicating that weight loss alone does not fully account for the likelihood of achieving comprehensive lipid improvement.

Importantly, a considerable proportion of women in the TRUE group achieved the Global Score despite modest weight loss at week 8, whereas many women in the FALSE group lost a substantial amount of weight without demonstrating corresponding improvements in lipid markers. This pattern, clearly visible in Figure 5, underscores weight loss and lipid improvement—although related—represent distinct physiological processes.

These findings suggest that improvements in lipid metabolism (e.g., reductions in triglycerides, LDL, or TC/HDL ratio) are influenced not only by the magnitude of weight loss but also by diet composition, hepatic fat oxidation, insulin sensitivity, and individual metabolic flexibility. Thus, meaningful lipid improvement can occur even with moderate weight reduction, highlighting the clinical importance of multi-marker evaluations over reliance on weight tracking alone.

### 3.8. J48 Decision Tree: Interpretable Prediction Rules

**Figure 6:**
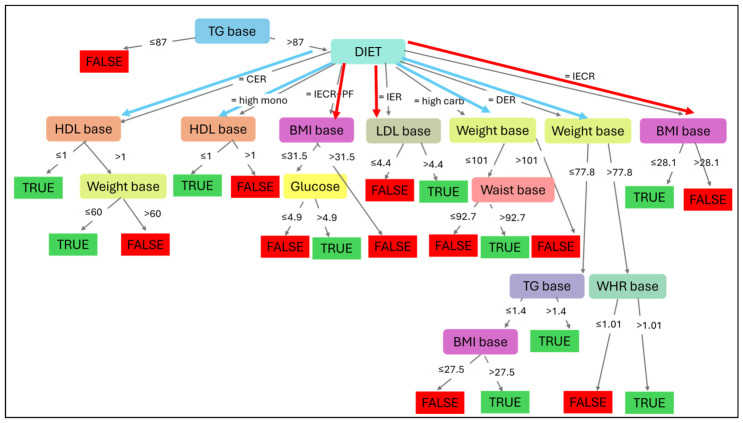
Hierarchical J48 Decision Tree Architecture for Global Score Prediction. The decision tree illustrates a multi-tier classification framework in which baseline triglyceride (TG) level serves as the primary root gatekeeper (≤0.87 vs. >0.87 mmol/L). For participants with baseline TG levels exceeding this threshold, the algorithm dynamically groups the seven dietary intervention modalities into two distinct, color-coded physiological pathways based on dietary strategy type. The red branches represent the three intermittent fasting variants (IER, IECR, and IECR+PF), whereas the blue branches represent the four traditional calorie-restricted and macronutrient-focused dietary regimens (ECR, CER, High Mono, and High Carb). Within the blue cluster, secondary partitioning is driven by baseline clinical variables including body weight, waist-to-hip ratio (WHR), total cholesterol (TC), and TG levels. Conversely, the intermittent fasting cluster (red branches) undergoes more complex secondary partitioning based primarily on lipid-related and metabolic markers, including baseline LDL, HDL, and BMI values. Rectangular leaf nodes denote the final classification outcomes (TRUE/FALSE) together with their corresponding case distributions.

To gain mechanistic insight into the predictors of Global Score outcome, the internal decision-making structure of the J48 classifier was examined. In this analysis, the diet type feature was added to the 11 baseline features. The classifier’s performance and structure were then evaluated through feature importance analysis and visualization of the final decision tree. Feature importance analysis identified five primary predictors of the Global Score outcome. Baseline triglycerides (TG) contributed the greatest predictive weight (45.42%), followed by the assigned dietary intervention (DIET, 24.02%), baseline LDL cholesterol (13.10%), baseline weight (11.35%), and baseline BMI (6.11%). The hierarchical structure of the J48 decision tree (Figure 6) directly reflects this ranking.

Root node. The tree’s root node partitioned participants by baseline TG (≤0.87 vs. >0.87 mmol/L). Women with TG ≤ 0.87 mmol/L were classified directly as FALSE (78 of 91 cases, 85.7%), demonstrating a physiological floor effect: an already-optimised lipid profile at baseline narrows the margin for further multi-marker improvement. This threshold (0.87 mmol/L, approximately 77 mg/dL) lies well within the standard optimal range (<1.7 mmol/L), indicating that it reflects limited remaining metabolic headroom rather than a pathological state. Women exceeding this threshold, where a greater capacity for lipid modification exists, were directed to a second split based on dietary intervention group.

Secondary split by dietary intervention. Among women with TG > 0.87 mmol/L, the tree partitioned participants into two conceptually distinct branches: intermittent restriction protocols (IER, IECR, IECR+PF), whose classification was governed by cardiovascular and metabolic markers, and continuous restriction and macronutrient-focused protocols (CER, High Mono, High Carb, DER), whose classification was governed primarily by baseline body composition (Figure 6).

Intermittent restriction pathways. Within the intermittent restriction arm, each protocol followed distinct decision logic. In the IER pathway, prediction relied on a cascade of cardiovascular markers: baseline LDL ≤ 4.4 mmol/L, further stratified by waist circumference ≤ 92.67 cm and HDL ≤ 1.3 mmol/L, predicted FALSE, whereas LDL > 4.4 mmol/L combined with age > 44 years and TG > 1.24 mmol/L predicted TRUE. In the IECR pathway, decision rules centred on anthropometric dimensions: weight > 77.8 kg combined with WHR > 1.01 predicted FALSE, while weight ≤ 77.8 kg predicted TRUE. In the IECR+PF pathway, classification integrated obesity and glycaemic boundaries: BMI ≤ 31.5 kg/m^2^ with fasting glucose ≤ 4.9 mmol/L predicted FALSE, while higher baseline glucose shifted the prediction to TRUE.

Continuous restriction and macronutrient-focused pathways. Within this arm, assignment to CER directly yielded a TRUE classification. For the remaining protocols, body mass was the primary determinant: baseline weight ≤ 71.9 kg predicted TRUE, while weight > 71.9 kg predicted FALSE.

Clinical implications. The divergent branching patterns across the two arms indicate that the clinical predictors governing lipid response differ substantially by dietary protocol type. These findings support the development of treatment-specific, personalised prediction models that account for individual baseline metabolic status rather than applying universal clinical thresholds.

## 4. Discussion

This study applied ML classification to pooled individual participant data from three RCTs to identify baseline clinical predictors of comprehensive lipid improvement across seven dietary interventions in overweight and obese women. Several key findings emerged. First, the composite Global Score—requiring simultaneous improvement across all four lipid-related parameters—was achieved by only 32.3% of participants, underscoring the physiological complexity of achieving broad lipid benefit. Second, macronutrient-focused interventions (High Mono and High Carb) consistently outperformed energy-restriction protocols on the Global Score. Third, baseline TG emerged as the single most influential predictor of response, followed by dietary intervention type, LDL, weight, and BMI. Fourth, age ≥ 45 years and intermediate obesity (BMI 30–35 kg/m^2^) were associated with higher rates of comprehensive improvement, while weight loss and lipid improvement were related but distinct outcomes. Taken together, these findings highlight the multifactorial nature of lipid response to dietary intervention and support the development of treatment-specific, personalized prediction models.

### 4.1. Lipid Improvement Rates and the Global Score

The overall Global Score rate of 32.3% reflects the stringency of requiring simultaneous improvement across four mechanistically distinct lipid parameters. Non-HDL cholesterol was the most frequently improved marker (65.8%), consistent with its sensitivity to energy-restricted diets, which reliably reduce apolipoprotein B-containing particles through hepatic fat clearance and reduce lipoprotein synthesis [8]. In contrast, LDL/HDL ratio was the least frequently improved parameter (50.0%), likely because LDL and HDL often respond in the same direction to energy restriction—with weight loss reducing both, thereby limiting net ratio improvement [43]. The relatively lower rates for TC/HDL and LDL/HDL ratios highlight the importance of selecting multi-marker composite outcomes that capture distinct dimensions of cardiovascular risk rather than relying on any single lipid parameter.

The superiority of High Mono and High Carb interventions on the Global Score (both 48.4%) is consistent with prior evidence that macronutrient composition, independent of energy restriction, modulates lipid profiles [19,22]. High-carbohydrate diets have been associated with preferential reductions in LDL and non-HDL cholesterol, while high-monounsaturated-fat diets favorably influence TG and HDL [44,45]. The observed pattern—High Carb leading on Non-HDL improvement (87.1%), High Mono on TC/HDL and LDL/HDL—suggests that these two macronutrient strategies engage complementary lipoprotein pathways, which may contribute to their shared superiority on the composite outcome, though causal attribution is not possible in the current design. In contrast, DER produced the lowest rates across all individual scores, possibly reflecting insufficient metabolic stimulus under a modest (~20%) daily energy deficit within the 12-week observation window. The differential efficacy of High Mono and High Carb diets relative to energy-restriction protocols is unlikely to be fully explained by direct hepatic effects alone, and emerging evidence implicates the gut microbiome as a critical intermediary in the diet–lipid relationship [37]. Dietary fat quality and carbohydrate content exert distinct and complementary effects on the intestinal microenvironment, reshaping microbial community structure in ways that downstream influence systemic lipid metabolism.

High-monounsaturated-fat diets have been associated with selective expansion of Lachnospiraceae—a family of butyrate-producing Firmicutes whose relative abundance correlates inversely with circulating LDL cholesterol and TG [46]. Lachnospiraceae ferment-resistant carbohydrates and dietary fibre to produce short-chain fatty acids (SCFAs), principally butyrate and propionate. Propionate acts as a substrate for hepatic gluconeogenesis while simultaneously suppressing de novo lipogenesis through inhibition of acetyl-CoA carboxylase, thereby reducing VLDL secretion and lowering circulating TG [47]. Butyrate, in addition to its role as the primary colonocyte energy substrate, activates intestinal farnesoid X receptor (FXR) signalling, which suppresses hepatic bile acid synthesis and reduces the enterohepatic cycling of cholesterol-derived bile acids—a key regulatory step in LDL clearance [48]. These microbial-mediated pathways represent plausible candidate mechanisms that could contribute to the advantage of High Mono diets on TC/HDL and LDL/HDL improvement observed in the present study (67.7% and 64.5%, respectively), though direct mechanistic evidence from the current dataset is lacking and causal attribution is not possible from the present observational design.

High-carbohydrate diets, by contrast, alter bile acid reabsorption patterns through a complementary pathway. Increased fermentable carbohydrate availability promotes expansion of Bifidobacterium and Lactobacillus species, which deconjugate primary bile acids via bile salt hydrolase activity [49]. Deconjugated bile acids are less efficiently reabsorbed in the ileum, resulting in greater faecal bile acid excretion, compensatory upregulation of hepatic CYP7A1—the rate-limiting enzyme in bile acid synthesis from cholesterol—and a consequent reduction in hepatic cholesterol availability and LDL secretion [48,49]. This mechanism is consistent with—though not demonstrated by—the pronounced advantage of High Carb diets on non-HDL cholesterol improvement observed here (87.1%), as LDL and VLDL particles are the primary substrates for hepatic cholesterol recycling via the bile acid pathway. Taken together, these complementary microbiome-mediated mechanisms—SCFA-driven suppression of hepatic lipogenesis for High Mono, and bile acid deconjugation-driven cholesterol clearance for High Carb—provide a mechanistic framework that reconciles the shared superiority of these two macronutrient strategies on the Global Score despite their distinct lipid-specific profiles.

### 4.2. Baseline TG as the Primary Predictor

The identification of baseline TG as the dominant predictor (45.42% of J48 feature importance; root node threshold 0.87 mmol/L) is consistent with the physiological role of TG as a sensitive marker of hepatic lipid flux, insulin sensitivity, and metabolic flexibility [50] and with evidence showing that different lipid fractions arise from distinct metabolic pathways [28]. The floor effect observed at this threshold—whereby women with already-optimized TG (≤0.87 mmol/L) were predominantly classified as FALSE—is clinically intuitive: individuals with a near-optimal lipid baseline have limited physiological headroom for further improvement within a fixed intervention period. This finding aligns with evidence from pharmacological trials demonstrating that baseline TG level is a key determinant of TG-lowering response [51] and extends this principle to dietary intervention contexts.

The threshold of 0.87 mmol/L (~77 mg/dL) is notably below the standard clinical cut-point for elevated TG (≥1.7 mmol/L), suggesting that even within the normal range, TG level discriminates metabolic responsiveness. This observation is hypothesis-generating: women with low-normal baseline TG may be less likely to achieve comprehensive lipid improvement, though this requires prospective confirmation before any clinical application, warranting alternative outcome targets or longer intervention periods. Conversely, women with TG > 0.87 mmol/L represent a subgroup with greater scope for multi-marker benefit, particularly when matched to an appropriately tailored dietary protocol.

### 4.3. Age and BMI as Modulators of Response

Women aged 45 years and older showed higher Global Score rates than younger women. However, these findings were derived from exploratory subgroup analyses and should be interpreted cautiously, given the limited sample size within some strata. This caution aligns with recent work showing that many precision-nutrition prediction models rely on high-dimensional omics and AI-enhanced dietary assessment tools, which limits their generalizability to routine clinical settings [30]. The observed associations may reflect biological differences, greater baseline lipid burden, or sampling variability, and require replication in independent cohorts. Perimenopausal hormonal transitions…may be associated with greater responsiveness to dietary lipid modification, though the cross-sectional nature of the age comparison precludes causal conclusions [9,52]. Additionally, older women in this sample may have had higher baseline lipid burden—providing more margin for improvement—consistent with the floor-effect logic identified by the J48 tree. The age threshold of 44 years, identified endogenously by the decision tree, aligns closely with typical perimenopause onset, reinforcing the biological plausibility of this stratification [53].

The non-linear relationship between BMI and Global Score success—with the highest rates in BMI 30–35 kg/m^2^ (40.0%), intermediate rates in BMI 25–30 kg/m^2^ (32.8%), and the lowest in BMI > 35 kg/m^2^ (22.7%)—suggests that moderate obesity may represent a zone of optimal metabolic flexibility. Women with BMI > 35 kg/m^2^ may experience chronic low-grade inflammation, hepatic steatosis, and impaired mitochondrial function that collectively blunt lipid responsiveness to short-term dietary modification [54]. These findings support a BMI-stratified approach to dietary lipid management, with more intensive or longer interventions potentially required for women with severe obesity.

To formally assess whether adiposity modulates age-related metabolic responsiveness, a multivariate logistic regression incorporating an Age × BMI interaction term was conducted (Table 8). The interaction term was statistically significant (OR = 0.992, 95% CI [0.984–1.000], *p* = 0.040), confirming that the relationship between age and dietary lipid responsiveness is not additive but depends on BMI level. BMI was independently and inversely associated with Global Score achievement (OR = 0.897, 95% CI [0.842–0.954], *p* < 0.001), whereas age alone was not a significant predictor (*p* = 0.168), indicating that its apparent effect is entirely contingent on adiposity status. Stratified success rates illustrate this interaction pattern clearly (Supplementary Table S3): among women with BMI 25–30 kg/m^2^, success rates increased markedly across age groups (23.1% in <40 years to 50.0% in >50 years), whereas among women with BMI >35 kg/m^2^, this age-related gradient was largely absent (20.8% to 27.3%). This pattern suggests that visceral adiposity may blunt the age-associated metabolic adaptations that otherwise favour dietary lipid responsiveness, possibly through mechanisms involving adipose tissue insulin resistance and impaired fatty acid oxidation capacity [54]. Notably, baseline TG was a significant positive predictor of Global Score (OR = 1.960, 95% CI [1.170–3.281], *p* = 0.011), likely reflecting the greater absolute improvement potential among women presenting with hypertriglyceridaemia at baseline—a finding consistent with regression to the mean and with the known sensitivity of TG to dietary carbohydrate and fat manipulation [50].

### 4.4. High Baseline Cholesterol and the Ceiling-to-Floor Principle

Women with high baseline total cholesterol (>6.0 mmol/L) achieved the Global Score at a substantially higher rate than those with normal values (46.2% vs. 28.3%), with the most pronounced effects for CER and IER (+38 percentage points). This observation underscores the importance of tailoring dietary strategies to baseline metabolic profiles, consistent with emerging frameworks for AI-driven personalization of food systems [27]. This pattern is consistent with a ceiling-to-floor principle: greater baseline elevation provides more absolute room for improvement, and energy-restricted diets reliably reduce hepatic cholesterol synthesis through upregulation of LDL receptor expression and reduced acetyl-CoA availability [55]. The absence of this effect in IECR+PF—where high-cholesterol women performed similarly or worse—may reflect the ad libitum fat content of this protocol, which could partially offset expected reductions in dietary cholesterol intake and blunt the lipid-lowering response.

However, several cholesterol-stratified subgroups contained fewer than 10–12 participants. Consequently, the observed differences may be sensitive to sampling variability and should be interpreted as exploratory observations rather than definitive subgroup effects.

### 4.5. Weight Loss and Lipid Improvement: Related but Distinct

The apparent decoupling between weight loss at Week 8 and comprehensive lipid improvement at Week 12—observed across all seven dietary groups and consistent with the weak overall correlation between weight change magnitude and Global Score (r = −0.163, *p* = 0.006)—suggests that caloric deficit alone is insufficient to predict favourable lipid remodelling, and that individual biological heterogeneity plays a substantial role. Several non-mutually exclusive mechanisms may explain why a significant proportion of women who lost weight (approximately 65–72% across energy-restriction protocols) failed to achieve comprehensive lipid improvement.

First, genetic variation in hepatic fatty acid oxidation capacity is a plausible contributor. Polymorphisms in genes encoding key regulators of mitochondrial beta-oxidation—including PPARA, CPT1A, and ACADM—have been associated with impaired lipid clearance during caloric restriction, such that some individuals fail to efficiently mobilise fatty acids released from adipose tissue into the hepatic oxidation pathway, leading to transient dyslipidaemia or absent lipoprotein remodelling despite negative energy balance [56]. Second, mitochondrial dysfunction—which is prevalent in women with severe obesity and type 2 diabetes risk—may limit the cellular capacity to oxidise the increased fatty acid flux that accompanies caloric restriction, diverting excess lipids toward re-esterification and VLDL secretion rather than clearance [57]. Third, pre-existing insulin resistance may uncouple adipose lipolysis from hepatic lipid metabolism: in insulin-resistant states, unrestrained lipolysis delivers excess free fatty acids to the liver regardless of dietary composition, potentially sustaining elevated LDL and TG even when caloric intake is restricted and body weight declines [58]. The notably lower decoupling rate observed in the High Carb and High Mono groups (52% versus 68–72% in energy-restriction protocols) is consistent with this interpretation, suggesting that macronutrient composition—rather than caloric deficit per se—may be a more effective lever for hepatic lipoprotein remodelling in this population.

With respect to the long-term trajectory of these effects, an important limitation of the current study is the absence of weight data beyond Week 8 and the absence of lipid data beyond Week 12, which prevents any assessment of whether the observed lipid improvements were sustained. This is clinically relevant because weight regain following short-term dietary intervention is well-documented [59], and the consequences for lipid profiles are predictable: restoration of adipose mass re-activates lipolysis-driven hepatic lipid flux, promotes re-elevation of VLDL and LDL, and rapidly reverses HDL gains achieved during weight loss [60]. The lagged or delayed nature of the lipid response—whereby lipid remodelling may continue to evolve for weeks after the primary weight-loss phase—is similarly unaccounted for in our 12-week observation window. Interventions of longer duration, or studies incorporating post-intervention follow-up lipid assessments at 6 and 12 months, are needed to determine whether the macronutrient-specific advantages observed here persist beyond the active intervention phase or are attenuated by rebound adiposity.

### 4.6. Machine Learning Performance and Model Selection

The LMT classifier achieved the strongest performance across all five metrics (AUC = 0.66, accuracy = 70%, recall = 0.70), though absolute discriminative ability was modest. The AUC range of 0.58–0.66 across models is comparable to that reported in other dietary response prediction studies using similar sample sizes and clinical feature sets [38,61], and should be interpreted in the context of the inherent difficulty of predicting individualized dietary response from baseline clinical variables alone. Non-clinical factors—including dietary adherence, gut microbiome composition, physical activity, and genetic variation—are likely to explain a considerable portion of the residual variance not captured by the 11 features used here [37,56]. Because formal statistical comparisons between classifier performances were not performed, the observed ranking of algorithms should be interpreted as descriptive rather than inferential. Consequently, although LMT achieved the numerically highest performance across the evaluated metrics, no conclusion can be drawn regarding its statistical superiority over the alternative classifiers.

To examine whether the composite Global Score obscured distinct metabolic trajectories, separate ML classifiers were trained for each individual lipid marker (LDL, HDL, total cholesterol, and TG). Unlike the Global Score where LMT was uniformly superior, the best-performing classifier varied by outcome (Supplementary Table S2A): Random Forest achieved the highest AUC for both LDL (AUC = 0.60, Accuracy = 73%) and TG improvement (AUC = 0.60, Accuracy = 72%), while LMT performed best for HDL (AUC = 0.70, Accuracy = 66%) and total cholesterol (AUC = 0.66, Accuracy = 79%). Overall, HDL improvement was the most predictable outcome, whereas LDL and TG proved the most challenging, suggesting that the baseline clinical feature set captures the inter-individual variability in HDL response more effectively than in other lipid fractions.

Feature importance profiles differed meaningfully across outcomes, consistent with the distinct physiological pathways governing each lipid parameter (Supplementary Table S2B). Baseline TG was the dominant predictor of TG improvement (importance = 0.08), consistent with its central role in hepatic de novo lipogenesis and VLDL secretion [50]. Baseline LDL ranked highest for the LDL improvement model (importance = 0.07), reflecting the direct dependence of LDL response on initial receptor-mediated clearance capacity. For HDL improvement, baseline HDL itself emerged as the strongest predictor (importance = 0.08), alongside waist circumference and hip circumference (both 0.06), consistent with the established inverse relationship between visceral adiposity and HDL metabolism. Total cholesterol improvement was most strongly associated with baseline total cholesterol (importance = 0.06), reflecting the shared hepatic LDL receptor clearance pathway with LDL. Taken together, these outcome-specific findings support the view that a universal composite endpoint, while clinically intuitive, may compress biologically meaningful heterogeneity across lipid subfractions and are consistent with the case for developing marker-specific predictive models for more targeted dietary prescriptions.

The choice of the J48 decision tree for in-depth interpretation, despite its lower AUC (0.58), is justified by its transparent, rule-based architecture. In clinical settings, an interpretable model that generates actionable decision rules—even at the cost of marginal predictive accuracy—may provide greater practical value than a higher-performing but opaque ensemble [57,62,63]. The branching structure of the J48 tree reinforces the interpretation that universal prediction models are likely to underperform relative to strategy-specific approaches.

Although the predictive performance was modest and insufficient for clinical deployment in its current form, the consistency of several predictors across multiple analytical approaches strengthens confidence in their potential biological relevance. Baseline triglycerides, body size measures, and cholesterol-related variables emerged repeatedly in Random Forest feature importance rankings, decision-tree structures, and subgroup analyses, suggesting that these predictors may capture meaningful signals despite the limited discriminative performance of the models. The moderate discriminative performance of all three classifiers—with AUC values ranging from 0.58 to 0.66—falls below the thresholds typically considered adequate for clinical decision support tools, where AUC values of ≥0.75–0.80 are generally required to justify integration into clinical practice guidelines [40]. An AUC of 0.66 indicates that the LMT model correctly ranks a randomly selected responder above a randomly selected non-responder in approximately two-thirds of cases—a meaningful improvement over chance, but insufficient to guide individual-level dietary prescriptions with confidence. Several structural barriers to clinical implementation further limit the translational potential of the current models: the models were developed and validated exclusively in overweight and obese women from three UK-based trials, limiting generalisability to other populations, sexes, and healthcare settings; the 11 baseline clinical features, while routinely available, were selected from a constrained pooled dataset rather than optimised through prospective feature engineering; and the binary Global Score outcome, while clinically intuitive, does not capture the magnitude of lipid change, which may be more clinically relevant than directional improvement alone. The present findings should therefore be interpreted as proof-of-concept evidence that ML-based stratification of dietary lipid response is feasible and hypothesis-generating, rather than as a validated clinical prediction tool.

### 4.7. Limitations and Future Directions

Several methodological limitations specific to the study design warrant explicit acknowledgement. First, the pooled sample of 284 participants across seven dietary intervention groups is modest relative to the complexity of the classification problem; per-intervention subgroup sizes ranged from 31 to 54, rendering treatment-specific models underpowered and all subgroup findings—including those related to age, BMI, and baseline cholesterol—hypothesis-generating rather than confirmatory. Second, significant baseline differences across intervention groups—in age, BMI, weight, and lipid profile—reflect the multi-trial pooling design and introduce residual confounding that ML feature adjustment cannot fully eliminate; between-group comparisons of lipid improvement rates should therefore be interpreted with caution. Third, the Global Score composite outcome was constructed specifically for this analysis and has not been independently validated as a clinical endpoint; although each component ratio captures a distinct and established dimension of atherogenic risk [5,6,7], the simultaneous improvement criterion is exploratory and requires prospective validation. Fourth, and most critically, all model performance estimates are based on internal 10-fold cross-validation; the absence of external validation in an independent cohort limits generalisability and means that the reported AUC values may overestimate true out-of-sample performance. External validation in a prospective, adequately powered cohort is a necessary prerequisite for clinical implementation. Additionally, the risk of overfitting cannot be excluded despite the use of stratified cross-validation, given the relatively small sample size relative to the number of predictors and intervention subgroups; independent external validation remains the only definitive test of true model generalisability.

Weight data were only available to Week 8, limiting the integration of weight change as a dynamic predictor of lipid response. Waist circumference, hip circumference, and WHR were available at Week 12 and are reported in Table 3; however, the absence of Week 12 weight data precluded a fully integrated body composition analysis at the primary endpoint. The outcome variable, defined at Week 12, may not capture longer-term lipid trajectories. Critically, the absence of post-Week-12 follow-up data prevents any assessment of whether the lipid improvements observed were sustained. Weight regain following short-term dietary intervention is well-documented, and restoration of adipose mass predictably re-activates lipolysis-driven hepatic lipid flux, promoting re-elevation of VLDL and LDL and rapid reversal of HDL gains [59]. Future studies should incorporate post-intervention lipid assessments at 6 and 12 months to determine whether the macronutrient-specific advantages observed here persist beyond the active intervention phase. Dietary adherence was not quantified through objective biomarkers—such as plasma carotenoids, fatty acid profiles, or urinary nitrogen—and actual dietary intake during the intervention period was not assessed, which prevents distinguishing true biological variability in metabolic responsiveness from differential adherence to the assigned dietary protocol. This limitation means that it is not possible to determine whether observed differences in lipid response across intervention groups reflect the intended macronutrient manipulation or variation in dietary compliance. Furthermore, socioeconomic factors—including educational attainment, food access, and income level—and behavioural characteristics such as stress, sleep quality, and smoking status were not available in the pooled dataset, despite their established influence on dietary adherence and lipid metabolism; their absence represents an additional source of unmeasured confounding. The analysis was restricted to women, and findings may not generalise to men or to populations with different baseline metabolic risk profiles. Formal statistical comparisons between classifier performances were not performed; consequently, the apparent superiority of the LMT model should be interpreted descriptively rather than as evidence of statistically significant differences between algorithms [63]. Additionally, the absence of genetic data—including PPARA polymorphisms and CPT1A variants [56]—gut microbiome profiling, and direct measures of insulin sensitivity [58] and mitochondrial function [57] limits the mechanistic interpretation of the decoupling observed between weight loss and lipid improvement, and represents a priority domain for future investigation.

Future research should seek to validate these prediction models in larger, prospective datasets and incorporate additional predictor domains including genetic variants such as APOE genotype and PCSK9 polymorphisms, gut microbiome composition [37], dietary adherence biomarkers, and physical activity assessed through objective measures such as accelerometry. The integration of sex-hormone status—particularly menopausal transition stage—may further improve prediction in this population, which has historically been underrepresented in cardiovascular risk models. Multi-trial collaborative platforms with harmonized data collection protocols would substantially increase statistical power and enable the development of more robust, externally validated treatment-specific prediction tools.

### 4.8. Future Perspectives

The present study establishes a proof-of-concept framework for ML-based prediction of dietary lipid response, and points to several promising directions for future research.

First, external validation in independent, multicentre cohorts is the most immediate and critical next step. The predictive models developed here were trained and evaluated exclusively on pooled data from three UK-based trials; their generalisability to other populations, ethnicities, healthcare settings, and dietary cultures remains untested. Prospective validation studies with pre-specified outcome definitions and adequate statistical power will be required to establish whether the identified predictors and decision rules replicate across diverse clinical contexts.

Second, the integration of multi-omics data—including genomics, metabolomics, proteomics, and gut microbiome profiling—has the potential to substantially improve predictive capacity beyond what is achievable with clinical variables alone [37,46,47,48,49]. Genomic variants such as APOE genotype and PPARA polymorphisms modulate individual lipid metabolism and dietary fat response [56]; plasma metabolomics profiles capture real-time substrate flux that baseline clinical variables cannot reflect; and gut microbiome composition mediates the diet–lipid axis through SCFA production and bile acid remodelling, as discussed in Section 4.1. The systematic integration of these data layers within a unified ML framework represents a natural extension of the current analytical approach.

Third, the development of dynamic, longitudinal models that incorporate temporal changes in body weight, dietary adherence, and metabolic biomarkers over the course of an intervention would address a fundamental limitation of the current static, baseline-only prediction framework. Recurrent neural networks, long short-term memory models, and other time-series architectures are well-suited to this task and could capture the trajectory of lipid response rather than its binary endpoint, potentially identifying early intervention signals that allow adaptive dietary modification mid-course.

Fourth, the integration of ML-based prediction with digital nutritional monitoring platforms—including wearable metabolic sensors, continuous glucose monitors, and AI-powered dietary tracking applications—represents a promising pathway toward real-time, adaptive dietary recommendations [27,30]. Such platforms could enable personalised nutrition prescriptions that update dynamically in response to an individual’s evolving metabolic state, moving beyond the static snapshot captured at baseline and toward a continuously learning clinical decision support system.

Fifth, and most importantly for demonstrating clinical utility, prospective randomised controlled trials are needed to evaluate whether ML-guided dietary prescriptions actually improve lipid outcomes compared with conventional, guideline-based recommendations. Without evidence from prospective RCTs, the clinical value of these predictive tools—however internally consistent—remains speculative. Such trials should incorporate pre-specified composite lipid endpoints, objective dietary adherence monitoring, and long-term follow-up to capture both the sustainability of lipid improvements and the risk of post-intervention rebound.

## 5. Conclusions

Comprehensive lipid improvement across multiple markers was achieved by approximately one-third of overweight and obese women enrolled in seven dietary interventions, with macronutrient-focused strategies—particularly High Mono and High Carb—consistently outperforming energy-restriction protocols. Machine learning analysis suggested that baseline TG level, dietary intervention type, LDL cholesterol, body weight, and BMI may serve as clinically informative predictors of response, and indicated that the decision rules governing lipid improvement may differ substantially across dietary protocol types—though these patterns require replication in larger, prospective datasets before firm conclusions can be drawn. A significant Age × BMI interaction (*p* = 0.034) indicated that the age-related advantage in lipid responsiveness is attenuated at higher levels of adiposity. The decoupling between weight loss and lipid improvement—observed in approximately two-thirds of participants across energy-restriction protocols—underscores that caloric deficit alone is an insufficient predictor of favourable lipid remodelling, and highlights the need for mechanistic studies incorporating genetic, mitochondrial, and insulin sensitivity profiling.

It is important to acknowledge that the predictive models demonstrated only modest discriminative ability (LMT AUC = 0.66), were evaluated through internal cross-validation only, and have not been tested in an independent cohort. These findings should therefore be interpreted as preliminary, hypothesis-generating evidence that ML-based stratification of dietary lipid response is feasible, rather than as a validated clinical prediction tool. Prospective external validation in independent cohorts, improvement of model discriminative performance, and integration of broader biological predictors—including genetic variants, gut microbiome composition, and objective dietary adherence measures—are necessary prerequisites before these findings can be considered for clinical translation.

## Figures and Tables

**Table 1:** (**A**) Baseline Anthropometric Characteristics by Dietary Intervention Group (n = 284). (**B**) Baseline Biochemical Characteristics by Dietary Intervention Group (n = 284).

(**A**)									
**Variable**	**CER** **(n = 53)**	**DER** **(n = 40)**	**IECR** **(n = 37)**	**IECR+PF** **(n = 38)**	**IER** **(n = 54)**	**High** **Carb** **(n = 31)**	**High** **Mono** **(n = 31)**	**Overall** **(n = 284)**	***p***
Age (years)	40.1 ± 4.1	47.9 ± 7.7	45.6 ± 8.3	48.6 ± 7.3	40.0 ± 4.0	46.9 ± 9.9	47.1 ± 10.3	44.5 ± 8.0	<0.001
Weight (kg)	81.5 ± 14.4	86.0 ± 16.7	79.4 ± 14.2	82.4 ± 15.7	84.4 ± 17.2	93.2 ± 14.2	92.8 ± 12.4	85.0 ± 15.8	<0.001
BMI (kg/m^2^)	30.8 ± 5.0	32.2 ± 5.6	29.6 ± 4.1	31.0 ± 5.7	30.6 ± 5.1	34.7 ± 4.3	35.0 ± 3.9	31.7 ± 5.2	<0.001
Waist (cm)	101.5 ± 13.4	106.0 ± 13.0	100.5 ± 11.8	104.1 ± 15.4	102.5 ± 13.8	101.3 ± 9.0	102.1 ± 9.6	102.6 ± 12.7	0.519
Hip (cm)	110.8 ± 10.1	112.6 ± 10.8	108.8 ± 8.8	111.0 ± 11.1	111.6 ± 11.3	121.1 ± 10.7	122.8 ± 8.9	113.4 ± 11.3	<0.001
WHR	0.91 ± 0.06	0.94 ± 0.06	0.92 ± 0.06	0.94 ± 0.07	0.92 ± 0.06	0.84 ± 0.06	0.83 ± 0.06	0.90 ± 0.07	<0.001
(**B**)									
**Variable**	**CER** **(n = 53)**	**DER** **(n = 40)**	**IECR** **(n = 37)**	**IECR+PF** **(n = 38)**	**IER** **(n = 54)**	**High** **Carb** **(n = 31)**	**High** **Mono** **(n = 31)**	**Overall** **(n = 284)**	***p***
HDL (mmol/L)	1.46 ± 0.30	1.35 ± 0.31	1.46 ± 0.42	1.45 ± 0.32	1.55 ± 0.39	1.51 ± 0.38	1.58 ± 0.30	1.48 ± 0.35	0.096
Total Chol (mmol/L)	5.15 ± 0.87	5.32 ± 1.03	5.25 ± 0.94	5.73 ± 1.21	5.19 ± 0.86	5.52 ± 0.75	5.38 ± 0.92	5.34 ± 0.96	0.074
LDL (mmol/L)	3.13 ± 0.75	3.34 ± 0.86	3.31 ± 0.88	3.74 ± 1.15	3.06 ± 0.84	3.19 ± 0.83	3.02 ± 0.84	3.25 ± 0.90	0.006
TG (mmol/L)	1.21 ± 0.65	1.19 ± 0.50	1.10 ± 0.56	1.17 ± 0.53	1.27 ± 0.65	1.51 ± 0.46	1.44 ± 0.60	1.26 ± 0.59	0.043
Glucose (mmol/L)	4.77 ± 0.37	4.98 ± 0.46	4.85 ± 0.46	4.96 ± 0.42	4.77 ± 0.47	5.07 ± 0.52	5.03 ± 0.57	4.90 ± 0.47	0.009

Values are mean ± SD. *p*-values from one-way ANOVA. Yellow shading: significant between-group difference (*p* < 0.05). CER, continuous energy restriction; DER, daily energy restriction; IECR, intermittent energy and carbohydrate restriction; IECR+PF, IECR with ad libitum protein and fat; IER, intermittent energy restriction; BMI, body mass index; WHR, waist-to-hip ratio. HDL, high-density lipoprotein; LDL, low-density lipoprotein; TG, triglycerides. For dietary group abbreviations, see Table 1A.

**Table 2:** Improvement rates (%) for individual lipid scores and composite Global Score, stratified by dietary intervention.

Diet (n)	TC/HDL	LDL/HDL	Non-HDL	TG/HDL	Global Score
High Mono (31)	67.7%	64.5%	80.6%	67.7%	48.4%
High Carb (31)	61.3%	58.1%	87.1%	67.7%	48.4%
CER (53)	50.9%	49.1%	58.5%	52.8%	30.2%
IECR (37)	56.8%	51.4%	56.8%	59.5%	24.3%
IER (54)	48.1%	44.4%	72.2%	50.0%	29.6%
IECR+PF (38)	44.7%	42.1%	63.2%	55.3%	28.9%
DER (40)	47.5%	47.5%	50.0%	45.0%	25.0%

TC, total cholesterol; LDL, low-density lipoprotein; HDL, high-density lipoprotein; TG, triglycerides; Non-HDL, non-HDL cholesterol; CER, continuous energy restriction; IER, intermittent energy restriction; IECR, intermittent energy and carbohydrate restriction; PF, protein and fat; DER, daily energy restriction.

**Table 3:** Changes in Anthropometric Measures from Baseline to Week 12 by Dietary Intervention Group (n = 284).

Dietary Group	n	Waist Circumference (cm)			Hip Circumference (cm)		
		Baseline	Change (Week 12)	*p* (Within)	Baseline	Change (Week 12)	*p* (Within)
CER	53	101.5 ± 13.4	−5.00 ± 4.50	***	110.8 ± 10.1	−4.32 ± 3.17	***
DER	40	106.0 ± 13.0	−3.47 ± 4.18	***	112.6 ± 10.8	−3.30 ± 4.15	***
IECR	37	100.5 ± 11.8	−5.53 ± 5.55	***	108.8 ± 8.8	−4.32 ± 3.97	***
IECR+PF	38	104.1 ± 15.4	−4.75 ± 4.07	***	111.0 ± 11.1	−3.95 ± 2.97	***
IER	54	102.5 ± 13.8	−3.74 ± 4.29	***	111.6 ± 11.3	−3.69 ± 3.79	***
High Carb	31	101.3 ± 9.0	−6.74 ± 4.34	***	121.1 ± 10.7	−7.97 ± 3.58	***
High Mono	31	102.1 ± 9.6	−6.63 ± 4.47	***	122.8 ± 8.9	−8.63 ± 4.50	***
Between-group ANOVA: F(6,277) = 3.02, *p* = 0.007				Between-group ANOVA: F(6,277) = 11.65, *p* < 0.001			

Values are mean change ± SD from baseline to Week 12. *p* (within group): paired *t*-test for change from baseline. Between-group ANOVA tests for differences in mean change across the seven dietary groups. *** *p* < 0.001. Green shading indicates statistically significant within-group change. CER, continuous energy restriction; DER, daily energy restriction; IECR, intermittent energy and carbohydrate restriction; IER, intermittent energy restriction; PF, protein and fat.

**Table 4:** Waist-to-Hip Ratio (WHR) Changes from Baseline to Week 12 by Dietary Intervention Group.

Dietary Group	n	WHR Baseline	WHR Change (Week 12)	*p* (Within Group)	Between-Group ANOVA
CER	53	0.91 ± 0.06	−0.01 ± 0.03	ns	F(6,277) = 1.16, *p* = 0.331
DER	40	0.94 ± 0.06	−0.00 ± 0.04	ns	
IECR	37	0.92 ± 0.06	−0.01 ± 0.04	ns	
IECR+PF	38	0.94 ± 0.07	−0.01 ± 0.03	ns	
IER	54	0.92 ± 0.06	−0.00 ± 0.03	ns	
High Carb	31	0.84 ± 0.06	−0.00 ± 0.04	ns	
High Mono	31	0.83 ± 0.06	+0.01 ± 0.03	ns	

WHR = waist circumference/hip circumference. None of the within-group changes in WHR reached statistical significance (all *p* > 0.05). Between-group ANOVA: F(6,277) = 1.16, *p* = 0.331, indicating no significant difference in WHR change across dietary groups. ns, not significant.

**Table 5:** Performance metrics of machine learning classifiers evaluated by 10-fold cross-validation for prediction of comprehensive cholesterol improvement (Global Score) in 284 women.

Metric	J48 Decision Tree	LMT	Random Forest
AUC	0.58	0.66	0.62
Accuracy	65%	70%	68%
F1-score	0.65	0.67	0.65
Recall	0.65	0.70	0.68
Precision	0.65	0.68	0.65

AUC, area under the receiver operating characteristic curve; LMT, Logistic Model Tree. Best-performing values per metric are highlighted in green.

**Table 6:** Global Score rate (%) stratified by age group and baseline BMI category. Highest rates per column are highlighted in green.

	BMI 25–30	BMI 30–35	BMI > 35	Overall
Age ≤ 44	23.9%	32.1%	17.8%	24.8%
Age 45+	44.2%	51.4%	30.0%	42.9%
Overall	32.8%	40.0%	22.7%	32.3%

BMI, body mass index (kg/m^2^). Age threshold set at 44 years based on the J48 decision tree split node. Age threshold of 44 years derived from J48 decision tree node; Table 7 and Supplementary Table S3 present continuous age analysis using tertile groupings.

**Table 7:** Global Score rate stratified by baseline total cholesterol level (normal: ≤6.0 mmol/L; high: >6.0 mmol/L) for selected dietary interventions.

Diet	Normal Chol (≤6 mmol/L)	High Chol (>6 mmol/L)	n (High)	Absolute Diff
CER	24.4%	62.5%	8	+38.1 pp
IER	23.9%	62.5%	8	+38.6 pp
High Carb	45.0%	54.5%	11	+9.5 pp
High Mono	45.5%	55.6%	9	+10.1 pp
IECR+PF	30.8%	25.0%	12	−5.8 pp

Only interventions with ≥8 participants in the high-cholesterol subgroup are shown. pp, percentage points. Positive values indicate a higher rate in the high-cholesterol subgroup.

**Table 8:** Multivariate Logistic Regression: Predictors of Global Lipid Score with Age × BMI Interaction (n = 284).

Variable	OR	95% CI	*p*-Value	Significance
Main effects and interaction term				
Age (centered, per year)	1.028	0.989–1.068	0.168	ns
BMI (centered, per kg/m^2^)	0.897	0.842–0.954	0.001	***
Age × BMI interaction	0.992	0.984–1.000	0.040	*
TG baseline (mmol/L)	1.960	1.170–3.281	0.011	*
Total Cholesterol baseline	1.291	0.922–1.807	0.137	ns
Dietary intervention (reference: Low Fat)				
DER vs. Low Fat (ref.)	0.542	0.186–1.585	0.263	ns
IECR vs. Low Fat (ref.)	0.462	0.157–1.359	0.161	ns
IECR+PF vs. Low Fat (ref.)	0.552	0.190–1.605	0.275	ns
IER vs. Low Fat (ref.)	0.887	0.372–2.112	0.786	ns
High Carb vs. Low Fat (ref.)	2.221	0.793–6.224	0.129	ns
High Mono vs. Low Fat (ref.)	2.745	0.962–7.834	0.059	ns
Model fit: n = 284|Events = 92 (32.4%)|AIC = 340.2|Pseudo R^2^ = 0.116|LR test (Age × BMI interaction): X^2^(1) = 4.478, *p* = 0.034				

Age and BMI were mean-centered prior to computing the interaction term (mean Age = 44.5 years; mean BMI = 31.7 kg/m^2^). OR, odds ratio; CI, confidence interval; TG, triglycerides; BMI, body mass index; DER, daily energy restriction; IECR, intermittent energy and carbohydrate restriction; IER, intermittent energy restriction; PF, protein-focused. Significance: *** *p* < 0.001; * *p* < 0.05; ns, not significant. Green shading indicates statistically significant results (*p* < 0.05).

## Data Availability

The table containing the data of this research may be found at the following link: https://github.com/shulash/Cholesterol-and-Intermittent-Fasting/blob/main/Supplementary%20Table%201.csv (accessed on 20 May 2026). Additional data will be provided upon request to shulash@openu.ac.il.

## Supplementary Materials

The following supporting information can be downloaded at: https://www.mdpi.com/article/10.3390/nu18121974/s1, Table S1: Individual-level baseline and follow-up anthropometric, biochemical, and dietary intervention data for the 284 study participants included in the analysis.; Table S2A: ML Classifier Performance for Individual Lipid Outcomes (10-fold cross-validation, n = 284); Table S2B: Random Forest Feature Importance (Mean Decrease in Impurity) by Individual Lipid Outcome; Table S3. Global Lipid Score Success Rates (%) by Age and BMI Strata.
