# Supplementary material for: Machine Learning-Based Prediction of Comprehensive Lipid Response to Dietary Interventions in Overweight and Obese Women
Source: Nutrients. 2026 Jun 18;18(12):1974. doi: 10.3390/nu18121974 (PMC13304630; doi:10.3390/nu18121974)
Supplement: Supplementary file 1 [file nutrients-18-01974-s001.zip › Supplementary_Material.pdf]

Table S2 A. ML Classifier Performance for Individual Lipid Outcomes (10-fold cross-validation, n = 284)

Outcome	Classifier	AUC	Accuracy	F1	Recall	Precision
LDL	J48 Decision Tree	0.51	70%	0.6	0.7	0.5
	LMT	0.58	70%	0.6	0.7	0.6
	**Random Forest**	**0.6**	**73%**	**0.8**	**1**	**0.7**
HDL	J48 Decision Tree	0.59	59%	0.59	0.59	0.59
	**LMT**	**0.7**	**66 %**	**0.65**	**0.65**	**0.65**
	Random Forest	0.68	63%	0.63	0.63	0.63
Total Cholesterol	J48 Decision Tree	0.54	78%	0.7	0.78	0.68
	**LMT**	**0.66**	**79%**	**0.88**	**1**	**0.79**
	Random Forest	0.61	79%	0.88	1	0.79
TG	J48 Decision Tree	0.51	72%	0.63	0.72	0.62
	LMT	0.5	73%	0.85	1	0.73
	**Random Forest**	**0.6**	**72%**	**0.67**	**0.72**	**0.67**
Global Score	J48 Decision Tree	0.58	65%	0.65	0.66	0.63
	**LMT**	**0.66**	**70 %**	**0.67**	**0.7**	**0.68**
	Random Forest	0.62	68%	0.65	0.68	0.65

The outcome represents the target column, which defines the change in each feature from baseline to 12 weeks; an improvement is classified as 'True', whereas no improvement is classified as 'False'. Green shading indicates the best-performing classifier for each outcome. AUC, area under the receiver operating characteristic curve; LMT, Logistic Model Tree; LDL, low-density lipoprotein; HDL, high-density lipoprotein; TG, triglycerides.

Table S2 B. Random Forest Feature Importance (Mean Decrease in Impurity) by Individual Lipid Outcome

Feature	Global Score	LDL	HDL	Total Cholesterol	TG
Total Cholesterol	**0.06**	0.06	0.05	**0.06**	0.04
LDL	0.05	**0.07**	0.05	0.04	0.04
Weight	0.05	0.04	0.06	0.04	0.04
Age	0.05	0.04	0.05	0.04	0.04
TG	**0.06**	0.04	0.05	0.05	**0.08**
Waist	0.05	0.03	0.06	0.05	0.04
BMI	0.05	0.03	0.05	0.04	0.04
Hip	0.05	0.03	0.06	0.04	0.04
Glucose	0.05	0.02	0.04	0.04	0.03
HDL	0.04	0.03	**0.08**	0.05	0.04
WHR	0.05	0.02	0.04	0.03	0.03

All features are measured at baseline. Green shading indicates the highest importance value within each lipid outcome column. Values represent the mean decrease in impurity (or Gini importance) contributed by each baseline feature to the Random Forest model's predictive performance. BMI, body mass index; HDL, high-density lipoprotein; LDL, low-density lipoprotein; TG, triglycerides; WHR, waist-to-hip ratio.

Table S3. Global Lipid Score Success Rates (%) by Age and BMI Strata

Age group	BMI 25–30 kg/m²		BMI 30–35 kg/m²		BMI > 35 kg/m²	
	n	Success %	n	Success %	n	Success %
< 40 years	26	23.1%	31	35.5%	24	20.8%
40–50 years	71	31.0%	40	**40.0%**	40	22.5%
> 50 years	22	**50.0%**	19	47.4%	11	**27.3%**

Values represent the percentage of participants achieving a Global Score of TRUE (simultaneous improvement in all four lipid parameters) within each Age × BMI stratum. Green shading indicates the highest success rate within each BMI column. Cell sizes range from n = 11 (>50y, BMI >35) to n = 71 (40–50y, BMI 25–30); small cell sizes should be interpreted with caution.
